# Across-species benefits of adrenalectomy on congenital generalized lipoatrophic diabetes: a review

**DOI:** 10.3389/fendo.2023.1151873

**Published:** 2024-01-08

**Authors:** Patricio H. Contreras, Pilar Vigil

**Affiliations:** ^1^ Reproductive Endocrinology Unit, Reproductive Health Research Institute, Santiago, Chile; ^2^ Endocrine and Gynecology Units, Fundación Médica San Cristóbal, Santiago, Chile

**Keywords:** Berardinelli-Seip syndrome, RU-486, Ketoconazole, adrenalectomy, adipose insulin resistance, anti-glucocorticoid therapy

## Abstract

Two adrenalectomies performed fourteen years apart notoriously alleviated insulin resistance in a female teenager with Congenital Generalized Lipoatrophy (CGL, 1988) and in a murine model of CGL (2002). Following a successful therapeutic trial with anti-glucocorticoids, we performed the first surgical procedure on an 18-year-old girl. Before surgery, the anti-glucocorticoid therapy produced a rapid and striking drop in fasting serum insulin levels (from over 400 to 7.0 mU/L) and a slower –but impressive– fall in fasting serum triglycerides from 7,400 to 220-230 mg/dL. In contrast, fasting serum glucose levels dropped more slowly, from 225-290 to 121-138 mg/dL. Two weeks following total adrenalectomy, the fasting serum glucose level was 98 mg/dL, with a corresponding serum insulin level of 10 mU/L. During an Oral Glucose Tolerance Test, the 2-hour serum glucose was 210 mg/dL, and serum insulin values during the test did not exceed 53 mU/L. In 2002, the A-ZIP/F1 hypoleptinemic mouse had its adrenal glands removed. Even though this CGL model does not respond well to leptin replacement, an infusion of recombinant leptin reduced the characteristic hypercorticosteronemia of this murine model of CGL. Adrenalectomy in this transgenic mouse improved insulin sensitivity in the liver and muscle. In summary, adrenalectomy –in both a human and a mouse case of CGL– limited adipose tissue exposure to corticosteroid action and led to a notorious metabolic improvement. On a broader scenario, given that leptin restrains the adrenal axis, the reduced leptin activity of the leptin resistance displayed by obese subjects should lead to adrenal axis overactivity. This overactivity should result in elevated serum levels of free cortisol, free fatty acids, and glycerol. In this manner, leptin resistance should lead to peripheral (adipose tissue, liver, and muscle) insulin resistance and islet beta-cell apoptosis, paving the way to Type 2 diabetes.

## 1 Introduction

Congenital Generalized Lipoatrophy (CGL, Berardinelli-Seip syndrome) is a rare autosomal recessive disorder with a prevalence of 1–10 patients per million people. Its low prevalence has led to limited clinical experience and a generalized therapeutic disappointment. The central finding of CGL is that a triglyceride-depleted adipocyte leads to a very severe case of insulin resistance. Indeed, it is much worse than that associated with a triglyceride-replete adipocyte (obesity).

There are at least four types of CGL (1): Type 1, secondary to mutations in the AGPAT2 gene located on chromosome 9q34 (encoding the enzyme 1-acylglycerol-3-phosphate O-acyltransferase 2); Type 2, secondary to mutations in the BSCL gene, located on chromosome 11q13 (encoding the enzyme Seipin); Type 3, due to mutations in CAV1 gene, found on chromosome 7q31 (encoding caveolin-1); Type 4, due to mutations in the PTRF gene, located on chromosome 7q21-2 (encoding cavin). There are also cases of CGL with unreported mutations.

About 95% of CGL patients will harbor either AGPAT2 or Seipin gene mutations. The commonest CGL is Type 1, and the rarest is Type 3. In Brazil and Japan, most CGL cases are Type 2, whereas in Europe, the Middle East, and Japan, most patients have CGL Type 1.

The extreme scarcity of fat depots in CGL subjects is associated with low circulating leptin and adiponectin levels. In contrast, these patients exhibit high levels of circulating insulin and triglycerides. Hypertriglyceridemia may induce a milky serum and cutaneous xanthomas. The lean organs of the CGL subjects will develop severe steatosis. Liver steatosis causes an enlarged, insulin-resistant organ, overproducing glucose (gluconeogenesis). Skeletal muscle steatosis produces a much-reduced ability to consume glucose in response to insulin. Fat-laden islet beta-cells are prone to enter apoptosis with a declining capacity to overproduce insulin. The above-described set of events usually leads to the development of diabetes by the second decade of life, requiring very high doses of exogenous insulin.

The current state-of-the-art treatment of CGL is subcutaneous recombinant leptin (2). However, there is limited accessibility to this recombinant hormone.

This review will discuss the therapeutic potential of negating corticosteroid action with adrenalectomy, anti-glucocorticoid therapy, or emerging ACTH blockers in managing CGL.

All these approaches might ameliorate the difficult metabolic situation of CGL subjects. Even though the information in this regard is quite limited, the findings –in a human case of CGL (3) and a murine model of the disease (4) –are highly encouraging.

## 2 Lipolysis and serum cortisol

At the basic level, lipolytic hormones are either catecholamines –epinephrine and norepinephrine– or natriuretic peptides. There is one critical antilipolytic hormone, insulin.

However, the whole picture is considerably more complex (5). Catecholamines may stimulate adenylyl cyclase, activating β1 and β2 adrenoreceptors to increase intracellular cAMP and induce lipolysis. At lower concentrations, catecholamines may inhibit adenylyl cyclase and lipolysis by activating α2 adrenoreceptors. Natriuretic peptides stimulate guanylyl cyclase to increase cGMP and lipolysis. cAMP stimulates PKA, while cGMP stimulates PKG. Both Protein Kinases phosphorylate Perilipin-1 and Hormone Sensitive Lipase (di-acylglycerol-lipase).

Cortisol and growth hormone (GH) are lipolytic hormones. They sensitize the adrenergic β1−β2 receptors and induce the angiopoietin-like protein 4 (ANGPTL-4). Its circulating level rises in response to fasting, hypoxia, and exercise, stimulating adenylyl cyclase to initiate the PKA lipolytic cascade. Hepatocytes and adipocytes synthesize ANGPTL-4. This protein blocks triglyceride clearance by inhibiting adipose tissue’s Lipoprotein Lipase (LPL). Studies in transgenic mice have shown that ANGPTL-4 overexpression decreased LPL activity and triglyceride clearance while increasing serum FFA and triglyceride levels. Cortisol enhances adrenergic-induced lipolysis, while total adrenalectomy reduces adrenergic-induced lipolysis.

At dawn, serum levels of cortisol reach their daily peak, and serum insulin levels reach their daily nadir. In addition, at this time of the day, serum ANGPTL-4 levels increase due to nightly fasting and increased cortisol levels. These circumstances are followed by acute lipolysis, translating into higher FFA and glycerol serum levels. FFAs act as fuels in the liver tissue and may be re-esterified to produce triglycerides. Glycerol stimulates hepatic gluconeogenesis to feed the brain when the hepatic glycogen content is exhausted. This picture strongly suggests a crucial lipolytic role for cortisol in fasting or stressful circumstances. In this regard, it is relevant to remember that Thomas Addison postulated in 1855 that the adrenal gland produces some substance that sustains life. In 1856, Charles Edward Brown-Séquard demonstrated that guinea pigs remained alive after unilateral adrenal removal and died after bilateral adrenalectomy. The life-sustaining ability of cortisol/corticosterone may depend on its capacity to rescue fat fuels (triglycerides) to feed the brain with glucose when liver glycogen is exhausted or when mammals face acute stress. The human brain accounts for about 2% of the body weight but takes about 20% of the glucose consumption. In other words, the brain is the primary consumer of glucose. Any situation limiting the brain’s availability of glucose is life-threatening.

On the other hand, Cushing’s syndrome displays limb subcutaneous fat atrophy, accompanied by central accumulation of fat (visceral fat and truncal fat). The latter suggests that permanent elevation of serum cortisol stimulates central and visceral lipogenesis. On the contrary, permanent hypocortisolemia (i.e., Addison’s disease) displays low body weight, suggesting reduced lipogenesis. Consequently, cortisol may act as a lipolytic hormone (during fasting and acute stress) or a lipogenic hormone during permanent glucocorticoid excess (Cushing’s syndrome, glucocorticoid therapy, and chronic stress).

## 3 Leptin-mediated metabolic amelioration in CGL patients

In 1998, Shimomura et al. (6) reported a remarkable metabolic amelioration in a murine model of a leptin-deficient CGL (the aP2-SREBP-1c mouse) treated with recombinant leptin. The authors of this paper concluded that insulin resistance was secondary to the severe hypoleptinemia found in these mice. These findings prompted Oral et al. (2) to start an open-label, prospective phase 2 study of leptin therapeutic potential on nine affected women (eight patients with CGL and one with FPLD) having a serum leptin level of less than 4 ng/mL.

These patients received subcutaneous Metreleptin. Eight out of nine women experienced a significant decrease in HbA1c levels. The insulin tolerance test showed amelioration of insulin resistance. Serum triglyceride levels decreased by about 60% after four months of Metreleptin therapy. The volume of the enlarged liver decreased by 28%, and the circulating levels of liver enzymes ALT and AST declined. Eight out of nine patients experienced weight loss secondary to a reduced liver size. After Metreleptin discontinuation, fasting serum insulin and triglycerides rose within 48 hours. The resumption of Metreleptin therapy promptly corrected this. Based on this –and subsequent studies– the FDA approved Metreleptin for the treatment of Generalized Lipodystrophies (https://www.accessdata.fda.gov/drugsatfda_docs/label/2014/125390s000lbl.pdf).

It is interesting to highlight that in CGL subjects, the serum levels of ANGPTL-3 (an LPL blocker) are elevated compared with control subjects. Metreleptin therapy in CGL subjects reduces hypertriglyceridemia, probably by inducing higher LPL action –triglyceride clearance– secondary to Metreleptin-induced reduction in serum ANGPTL-3 levels (7).

As expected, Metreleptin normalizes hyperphagia shortly after the initiation of treatment. However, a surprising effect was that Metreleptin therapy in Generalized Lipodystrophies improved insulin sensitivity independent of food intake: Brown et al. (8) demonstrated that Metreleptin improved muscle and hepatic insulin sensitivities in subjects with lipodystrophy. There was also a reduced fasting serum glucose and triglycerides and reduced liver fat content in CGL patients whose food intake was unchanged.

However, the mechanisms by which Metreleptin improves insulin sensitivity in Generalized Lipodystrophies (congenital and acquired) are still unclear. Nevertheless, leptin replacement is currently the state-of-the-art treatment in CGL patients (2). The potential advantage of disclosing the insulin-sensitizing mechanisms of Metreleptin is the replication of this effect with alternative, affordable, and easily usable therapeutic approaches. We have postulated (3) that leptin replacement restores a normal, leptin-mediated restrain of the adrenal axis in CGL. This mechanism limits adipocyte exposure to cortisol-driven lipolysis. If this hypothesis is true, any therapy limiting adipocyte exposure to cortisol might be helpful in the management of CGL. It is important to remember that rodents lacking leptin action (ob/ob and db/db mice) are hypercorticosteronemic (9). This fact supports the notion that leptin is necessary to restrain the adrenal axis. Besides, adrenalectomy in these mice notoriously ameliorates their insulin resistance (10).

The extreme adipocyte insulin resistance –characteristic of CGL– is critical in the induction of the severe steatosis of lean organs, producing this disorder’s grotesque insulin resistance characteristic. Let us assume that a high level of serum free-cortisol is crucial to overstimulating lipolysis. In this case, metabolic amelioration in CGL subjects might be feasible with various therapeutic approaches. The latter include anti-glucocorticoid therapies, ACTH blockers (currently in development), and total adrenalectomy, followed by a limited corticoid replacement. However, until now, there has been no demonstration in the medical literature of an overactive adrenal axis in human patients having CGL.

## 4 Adrenalectomy in a human case of CGL diabetes (1988)

We initially reported (11) a successful anti-glucocorticoid trial with Mifepristone-alone followed by Mifepristone plus Ketoconazole on a female teenager with Berardinelli-Seip Congenital Lipoatrophic Diabetes. We presented a poster at the Seventy-First Meeting of the Endocrine Society (1989). Its title, “*Successful reversal of the insulin resistance of Congenital Lipoatrophic Diabetes (CLD) by anti-glucocorticoid (AGC) therapy (RU486)*”, alluded to the extraordinary results of the anti-glucocorticoid trial that preceded the decision to perform total adrenalectomy on the patient. Over a year ago (March 16th, 2022), one of us (PHC) reported this case in extent (3). We must emphasize that we first met our patient in 1986, eight years before leptin came to light. Of course, the pathogenesis of CGL was cloudier at the time (36 years ago) compared with 2022.

In 1989, after the experience with the patient had ended, we could not explain our results due to the insufficient knowledge of adipocyte biology. This obstacle precluded a formal submission of our experience. The exponential expansion of our understanding of adipocyte biology allowed one of us (PHC) to elaborate on a rational explanation of our extraordinary findings (3).

After a year of therapeutic failures, in 1988, we decided to attempt an anti-glucocorticoid therapeutic trial on our 18-year-old patient with CGL diabetes. We suspected that unrestrained lipolysis was causing the empty-adipocyte syndrome in our patient. Moreover, we believed this excessive lipolysis was secondary to adipose tissue exposure to cortisol.

In summary, we hypothesized that serum cortisol induced a progressive triglyceride depletion of the adipocyte. The reason was simple: At that time, cortisol was regarded as necessary to stimulate the intracellular activity of the hormone-sensitive lipase (HSL or di-acylglycerol lipase).

The advantage of this daring hypothesis was its testability by using anti-glucocorticoid drugs. So, we initially treated our patient with Mifepristone (RU 486, a potent anti-glucocorticoid and anti-progesterone steroidal drug), 600 mg daily for nine weeks. However, by previous experience with the drug, we were aware that glucocorticoid antagonism would inevitably lead to compensatory higher serum ACTH and cortisol levels. This hypercortisolemia, in turn, would reduce Mifepristone action. To minimize the expected Mifepristone-induced hypercortisolemia and potentiate Mifepristone action, we added Ketoconazole, 800 mg daily, for one week. Finally, we gave Mifepristone alone, 600 mg daily, for two weeks to evaluate the influence of Ketoconazole withdrawal on serum insulin, glucose, and triglyceride levels. None of the extraordinary metabolic results of the anti-glucocorticoid trial (3) contradicted our hypothesis. All gathered evidence supported the idea that, by opposing cortisol action, the metabolic disorder improved notably.

**Figure 1:**
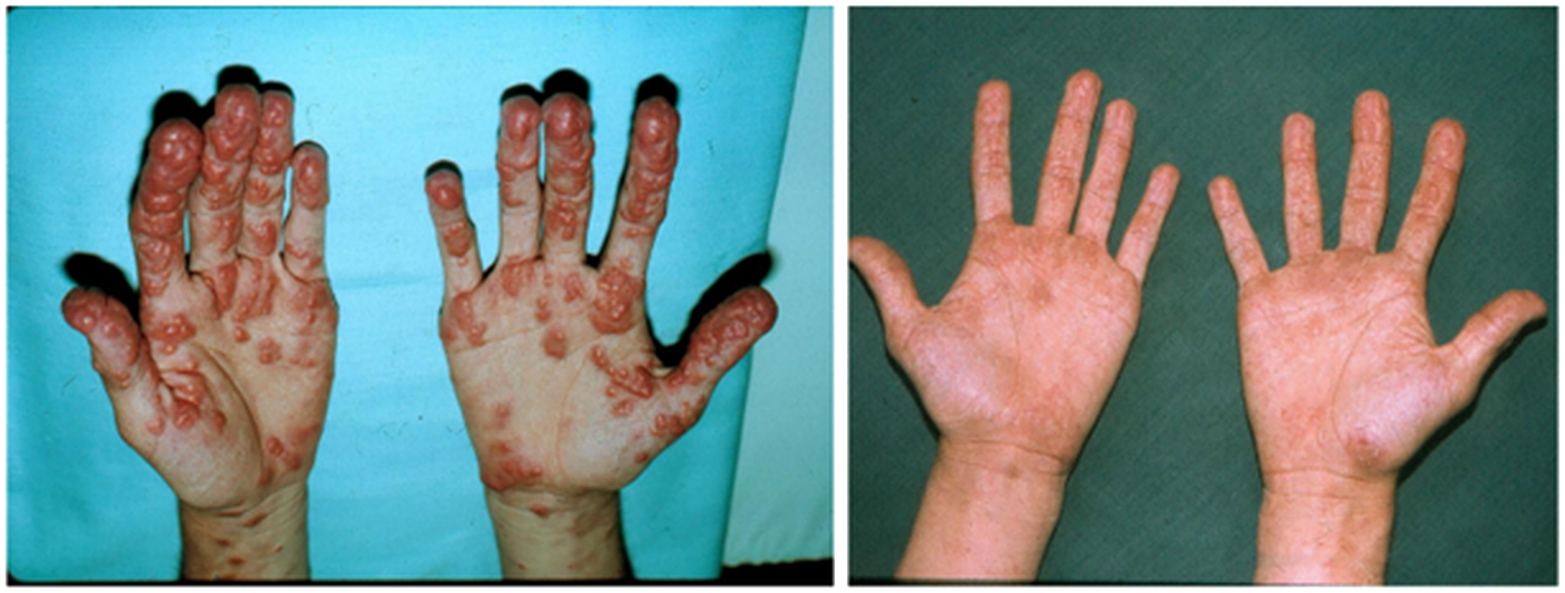
The eruptive xanthomas of the hand disappeared after 12 weeks of anti-glucocorticoid therapy.

**Figure 2:**
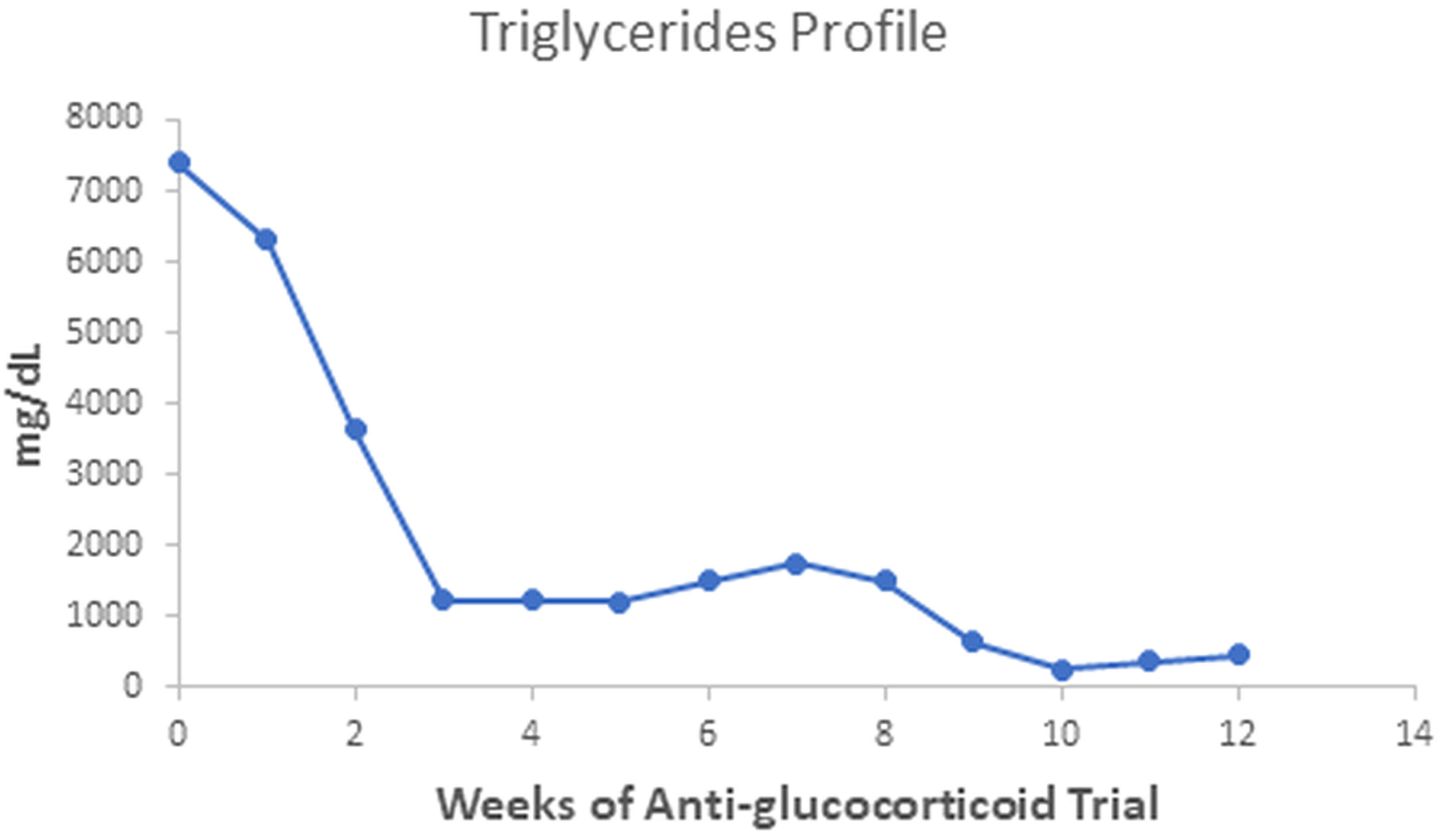
Serum triglycerides fell more slowly than insulin values during therapy with Mifepristone alone; when we added Ketoconazole, the levels achieved the minimal observed value. After Ketoconazole discontinuation, these values rose slightly.

**Figure 3:**
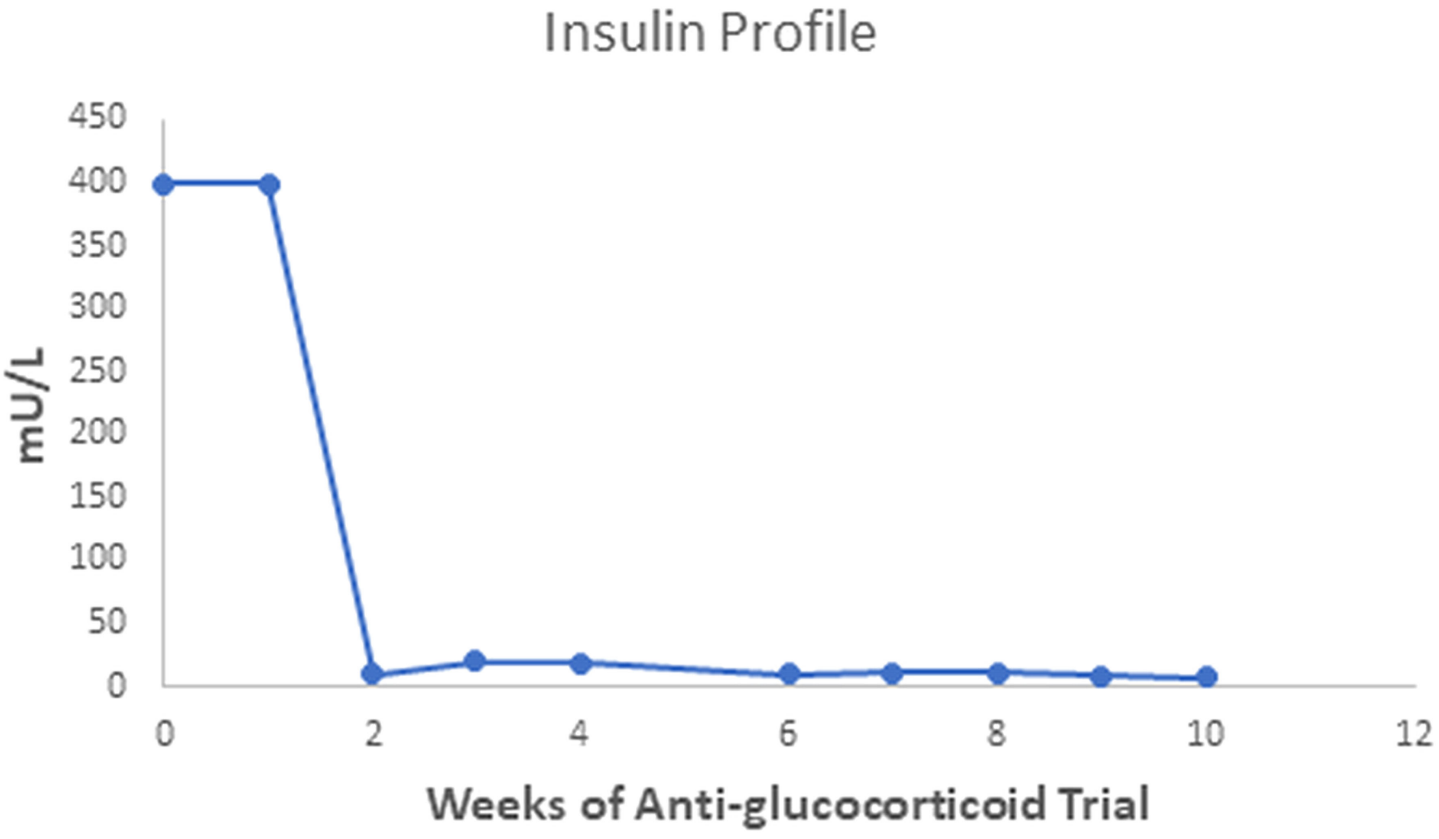
After two weeks of Mifepristone alone, the serum insulin level dropped from levels above 400 to between 10 and 20 mU/L; after we added Ketoconazole (tenth week), serum insulin fell to a minimum of 7 mU/L. The serum insulin value of the fifth week is not available.

**Figure 4:**
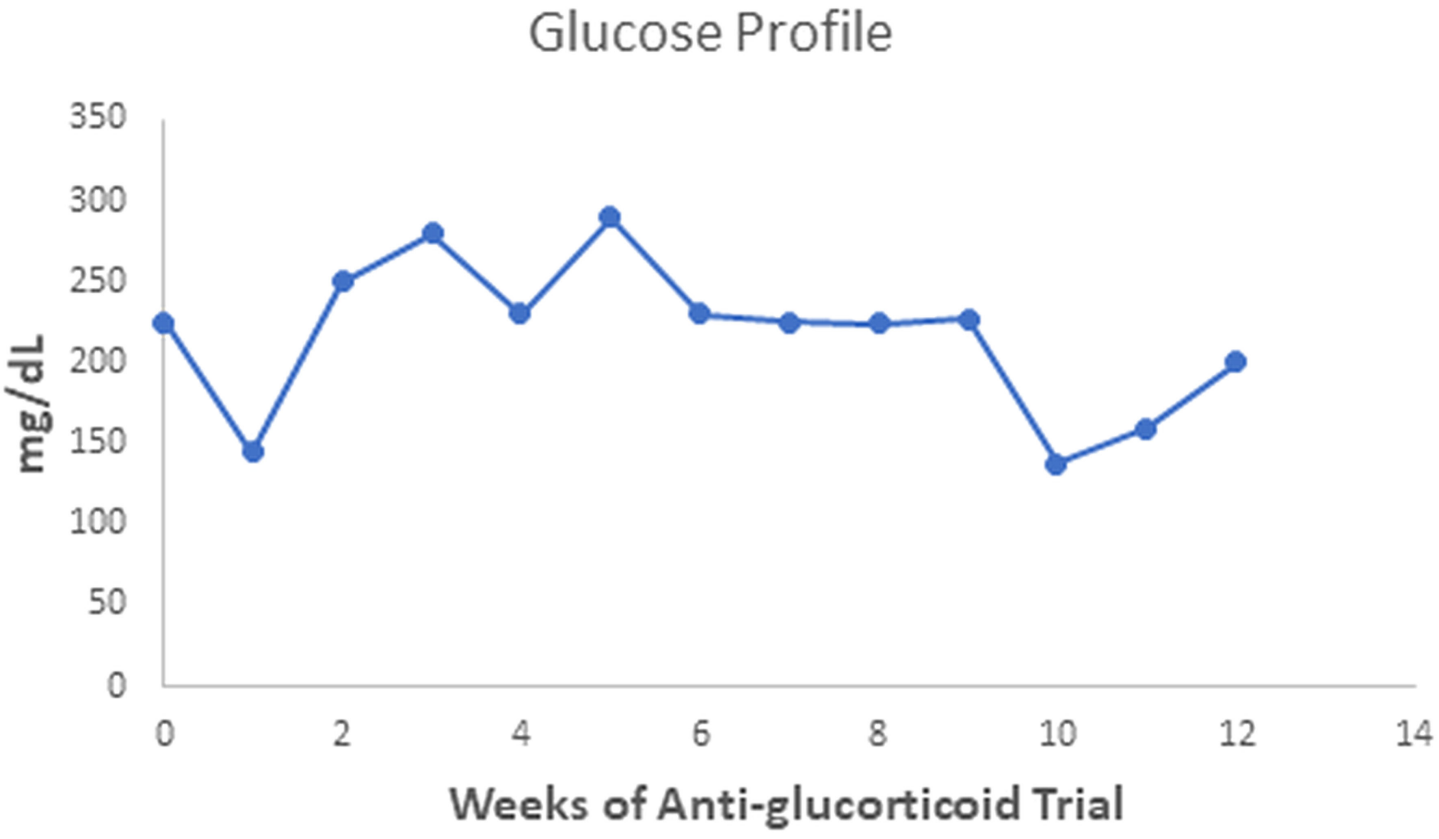
Serum glucose values oscillated during the anti-glucocorticoid trial. After a week with Ketoconazole, they achieved the lowest level (138 mg/dL), rising to 200 mg/dl two weeks after discontinuation.

The patient gained 7 kilograms, suggesting improved triglyceride storage in the adipocyte. Acanthosis nigricans and eruptive xanthomas disappeared within 12 weeks of anti-glucocorticoid intervention (**Figure 1** in reference 3 and **Figure 2** in this review). **Figures 2**-**4** show the evolution of serum insulin, triglycerides, and glucose during the anti-glucocorticoid treatment.

These encouraging results led to the elaboration of a second hypothesis: Since chronic treatment with Mifepristone was impossible, our data strongly suggested total adrenalectomy, followed by limited cortisol replacement with added fludrocortisone, might help our patient.

Total adrenalectomy was successful, and 24 hours following the surgical intervention, fasting serum glucose was within the normal range. The OGTT results showed a fasting serum glucose level within normal limits and a 2-hour serum glucose level of 210 mg/dL. Moreover, serum fasting insulin was just 10 mU/L, and the maximal serum insulin value during the OGTT was 53 mU/L. We discharged the patient medicated with daily hydrocortisone 15 mg (10 mg AM, plus 5 mg PM) and 0.1 mg fludrocortisone at 8 AM.

Nowadays, the patient is a 53-year-old married, lipoatrophic diabetic woman. She had two pregnancies, with one spontaneous abortion and one premature delivery (aged 33 and 34, respectively). Child delivery is exceptional among women affected with CGL, probably due to their severe hypoleptinemia. We suppose that her pregnancies were made possible by the fact that she constantly put on weight, so her serum leptin levels might have risen accordingly, allowing for the pro-reproductive actions of the hormone. Her weight-gaining process might be due to less exposure of her fatty tissue to cortisol. The reduced exposure of adipose tissue to cortisol was due to total adrenalectomy and limited cortisol replacement therapy.

She has fared relatively well this long despite never receiving leptin replacement. She currently weighs 72 kilograms (BMI 27.1), considerably more than the 52 kilograms she had at the anti-glucocorticoid trial. Nowadays, she receives high doses (260 Units) of regular and NPH insulin plus metformin (1,700 mg) and fenofibrate. Her fasting serum glucose levels oscillate from normal to high levels in fasting conditions, while her HbA1c level is 8.1%. She also receives hydrocortisone 15 mg plus fludrocortisone 0.1 mg at 8 AM to replace adrenal hormones. Serum triglycerides are 676 mg/dL (versus 7,400 mg/dL before the anti-glucocorticoid treatment). She has positive microalbuminuria, suggesting initial renal damage. Her liver has increased consistency and volume. A tetrapolar BIA revealed that her percent fat mass was just 6.2%. A more recent tetrapolar BIA gave a 9.1 percent fat mass. To ensure we had a correct figure for this parameter, we subjected our patient to a DEXA examination that revealed a 9.2 percent body fat with a BMI of 27.1.

In August 2022, the patient had a revealing complication. Her attending physician wanted to improve her metabolic control by adding Empagliflozin 12.5 mg a day (half a 25 mg tablet). The patient requested our opinion on the matter. We suggested to her that hypertriglyceridemia might worsen with Empagliflozin, given the expected rise in serum glucagon. As predicted, her serum triglycerides rose to 2,636 mg/dL after three weeks on Empagliflozin. At the hospital, she received high doses of pump-administered crystalline insulin. She also stopped receiving Empagliflozin. After a few weeks without Empagliflozin, her serum triglyceride level dropped to 680 mg/dL. We interpret these findings as a natural consequence of having lipolysis-prone fat tissue. Higher circulating lipolytic factors (such as glucagon) may enhance lipolysis and elevate serum triglyceride levels.

Given her clinical picture (such as her average intelligence and the presence of mechanical fat), we believed she had an AGPAT2 mutation. The attempts made by her current attending physicians to have her genetically sequenced in the US failed for over a decade. Ultimately, her genetic sequencing succeeded, finding a gene mutation, whose details remain classified (Drs. E. Oral and A. Garg, personal communication).

## 5 Adrenalectomy in a murine model of CGL (2002)

In 1998, Shimomura et al. (6) developed a murine model of CGL by overexpressing a truncated version of the sterol regulatory element-binding protein-1c (also named adipocyte determination and differentiation factor-1, ADD-1) in the adipose tissue. The truncated version of the SREBP-1c blocks adipocyte differentiation instead of enhancing it, as the natural protein does. Therefore, these mice exhibited a disordered differentiation of their adipose tissue, with a marked reduction in fat depots. They also exhibited marked hyperinsulinemia and severe hyperglycemia unresponsive to exogenous insulin. Liver steatosis was present at birth, and hypertriglyceridemia appeared later in life. Brown fat was hypertrophic. In contrast to the remaining murine models of insulin resistance, these animals were not obese and resembled human CGL subjects.

Later, Shimomura et al. reported in 1999 (12) that this hypoleptinemic murine model of CGL diabetes responded to murine recombinant leptin replacement with a significant metabolic amelioration. According to these investigators, the severe hypoleptinemia of these mice was responsible for their extreme insulin resistance, characteristic of their CGL diabetes. These authors recommended a clinical trial of leptin replacement in human CGL patients, given that they usually exhibit marked hypoleptinemia. As previously mentioned, this critical information led Oral et al. (2) to demonstrate a beneficial effect of leptin replacement in human lipodystrophy cases.

However, not any model of murine CGL responded well to leptin replacement. The A-ZIP/F1 mouse (13, 14) has lesser residual fat depots than the transgenic mouse developed by Shimomura et al. (6). Moitra et al. (14) developed this model in 1988. The relative therapeutic failure of leptin replacement in the A-ZIP/F1 mouse suggested that leptin deficiency was not the sole cause of insulin resistance in this murine model of CGL. However, leptin replacement reduced the elevated corticosterone level affecting these mice, suggesting that leptin restrains the adrenal axis. A double transgenic mouse model, the LepTg/+: A-ZipTg/+, overexpressing leptin in the liver tissue, showed a reversal of the hyperphagia and a reduction of the severe steatosis of the A-ZIPTg/+ mouse. They also displayed an improved insulin sensitivity, demonstrating the positive effect of leptin on the metabolic disarray of the A-ZIPTg/+ mouse (15).

In 2002, Haluzik et al. (4) tested the hypothesis that the elevated serum corticosterone of the A-ZIP/F1 mice contributed to their severe insulin resistance. Adrenalectomy in these mice resulted in improved hepatic and muscle insulin sensitivity. This result suggested that increased serum corticosterone levels (seven-fold) contributed to the diabetes of these mice. Therefore, insulin sensitivity in both muscle and liver improved by removing the glucocorticoid excess. Serum FFA did not fall significantly following total adrenalectomy (from 682 in sham-operated animals to 605 mmol/L in adrenalectomized A-ZIP/F-1 mice), suggesting *prima facie* that lipolysis did not fall with adrenalectomy. However, adrenalectomy substantially reduced serum insulin from 172 to 75 ng/mL. Since the product of FFA times insulin reflects adipose insulin resistance, the conclusion is that adrenalectomy in these mice was associated with a 61.3% reduction in adipose insulin resistance (lipolysis). Of course, this reasoning is only valid if the A-ZIP/F-1 mice retain some minimal functional adipose tissue.

In summary, both low serum leptin and high serum corticosterone levels contribute to the severe insulin resistance exhibited by these A-ZIP/F1 mice. These two contributors do not appear to act independently since leptin replacement reduces corticosterone levels (13). So, these findings suggest that serum leptin restrains the adrenal axis in this model of murine CGL.

## 6 Leptin restrains the adrenal axis

As mentioned, mice models with absent or reduced leptin action (ob/ob and db/db) are obese and insulin-resistant and display adrenal hyperplasia and elevated serum corticosterone levels (9). These facts suggest that leptin inhibits the adrenal axis, while hypoleptinemia leads to overactivity. Makimura et al., 2000 (16) reported that adrenal removal in leptin-deficient ob/ob mice reversed their obese phenotype and corrected their hypothalamic melanocortin tone. So, hypoleptinemia requires the presence of the adrenal gland to induce insulin resistance. In summary, we must find the intermediate steps between absent or insufficient leptin action and insulin resistance. In experimental animals with hypoleptinemia, the adrenal gland appears to mediate the development of insulin resistance.

In 2014, Perry et al. (17) showed that leptin reverses diabetes by suppressing the hypothalamic-pituitary-adrenal axis. In three rat models of poorly controlled diabetes, they showed that the elevated rates of liver glucose production were secondary to hypoleptinemia-induced adrenal cortex overactivity. This adrenal overactivity resulted in high rates of adipocyte lipolysis. Moreover, corticosterone infusion mimicked the effects of hypoleptinemia. In summary, leptin replacement in rat models of poorly controlled diabetes reduces lipolysis, liver glucose production, and hyperglycemia.

Later, Perry et al. (18) further studied the mechanism behind the leptin reversal of diabetic ketoacidosis. Leptin infusion –in a rat model of streptozotocin-induced diabetic ketoacidosis– reduced within 6 hours the rates of lipolysis, hepatic glucose production, and hepatic ketogenesis by 50%, independently of serum glucagon levels. These leptin effects disappeared through a co-infusion of corticosterone. Moreover, treating these rats with an adipose triglyceride lipase inhibitor blocked corticosterone-induced increases in serum glucose levels and rates of liver glucose production and ketogenesis. The positive effects of leptin replacement in CGL patients may be due to restraining the adrenal axis, which should reduce lipolysis.

The burden of ectopic fat in lean organs (steatosis of the liver, the muscle, and the islet beta-cell) falls by diminishing lipolysis. This latter effect should result in diminished liver and muscle insulin resistance and better beta-cell survival.

## 7 CGL as a model of grotesque adipocyte insulin resistance

The best clinical surrogate marker of adipose insulin resistance is the product of fasting serum insulin times fasting free fatty acids, FFA (19). So, estimating adipose insulin resistance is a relatively easy task in the clinical arena. The insulin concentration required to inhibit lipolysis by 50% (measured by the multistep pancreatic clamp) correlates strongly with the adipose insulin resistance index (19).

CGL subjects exhibit a remarkable elevation of serum insulin and a lesser elevation of serum FFA, so they have an extreme form of adipose insulin resistance. As stated, we have postulated that CGL subjects exhibit cortisol-mediated unrestrained lipolysis, leading to high serum FFA and glycerol levels (3). We did not have access to the measurement of FFA at the time of the anti-glucocorticoid trial in our patient, but we did measure serum triglyceride levels. This measurement allowed us to obtain an alternative estimate of adipose insulin resistance in our patient: the product of serum fasting triglycerides (mg/dL) times serum fasting insulin (mU/L). We named it the Adipo-IR-TG to distinguish it from the original surrogate, the Adipo-IR-FFA. A Japanese study (20) found that adipose tissue insulin resistance was related to elevated serum triglyceride levels and reduced HDL cholesterol levels. So, we used serum triglyceride instead of FFA values to estimate the adipose tissue insulin resistance (Adipo-IR-TG).

The opportunity to test our maneuver came in 2021: Zhang et al. (21) studied six sets of patients of both sexes: control subjects, obese subjects without metabolic syndrome, and obese subjects with metabolic syndrome. Each group of patients had their adipose insulin resistance index measured: serum fasting insulin (mU/L) times serum FFA (mmol/L). Also, they had their serum triglyceride level measured. We calculated the Adipo-IR-TG in these six groups of patients by multiplying their group average serum fasting insulin levels (mU/L) times their group average serum fasting triglyceride levels (mg/dL). We also calculated the Adipo-IR-FFA in these six groups of patients by multiplying their group average serum fasting insulin levels (mU/L) times their group average serum FFA levels (mmol/L). In this way, we had six pairs of both Adipo-IR indices to compare.

The correlation coefficient between the Adipo-IR-FFA and the Adipo-IR-TG was 0.993. Linear regression of these two Adipose-IR indices allowed us to predict the Adipo-IR-FFA index with the calculated Adipo-IR-TG figure.

The prediction equation is as follows:


Predicted Adipo−IR−FFA=1.651511032+(0.003192495*Adipo−IR−TG)

In other words, the predicted Adipo-IR-FFA value is approximately 3.2 percent of the Adipo-IR-TG value, plus 1.65. So, with an Adipo-IR-TG figure of 1,200 mU/L*mg/dL, one would expect an Adipo-IR-FFA value of 5.49 mU/L*mmol/L. The predictive power of this equation was surprisingly accurate since the equation relating the predicted Adipo-IR-FFA figure with the measured Adipo-IR-TG was very close to unity:


Predicted Adipo−IR−FFA=0.084+(0.986*Measured Adipo−IR−FFA)

In the study by Zhang et al., the upper limit for the Adipo-IR-FFA was 3.18 mU/L*mmol/L in control males and 3.63 mU/l*mmol/L in control females. With a serum insulin value of 6 mU/L and a serum triglyceride of 100 mg/dL, we obtain an Adipo-IR-TG figure of 600 mU/l*mg/dL and a predicted Adipo-IR-FFA value of 3.57 mU/L*mmol/L (a figure in between the upper limits of control men and women in Zhang’s study). An Adipo-IR-TG of 600 (mU/L*mg/dL) appears as a reasonable upper limit of the normal range for both sexes.

**Figure 5:**
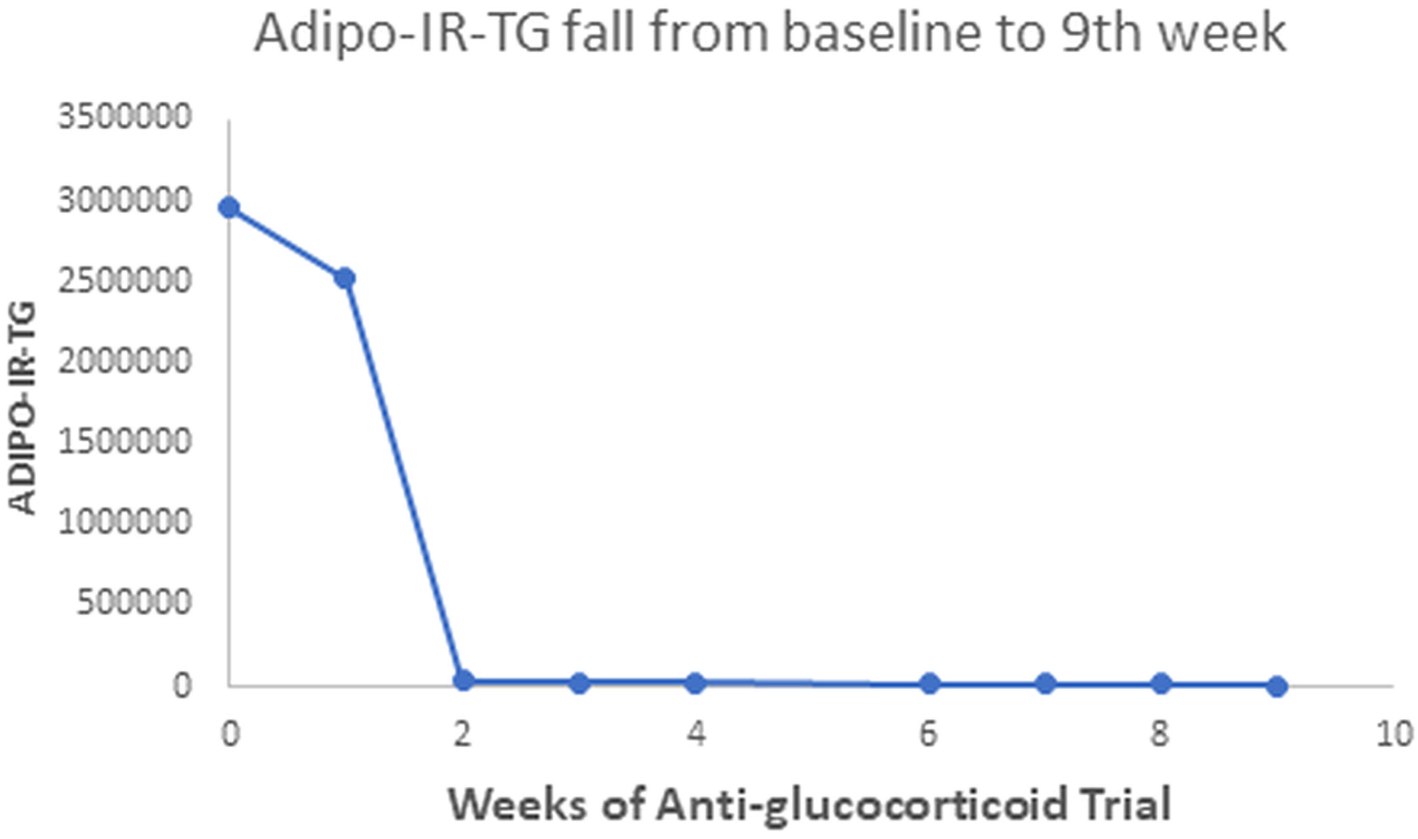
This Graph shows the fall in Adipo-IR-TG values from a sky-high baseline level of 2,960,000 to 4,936 mU/L*mg/dL at the end of the ninth week.

**Figure 6:**
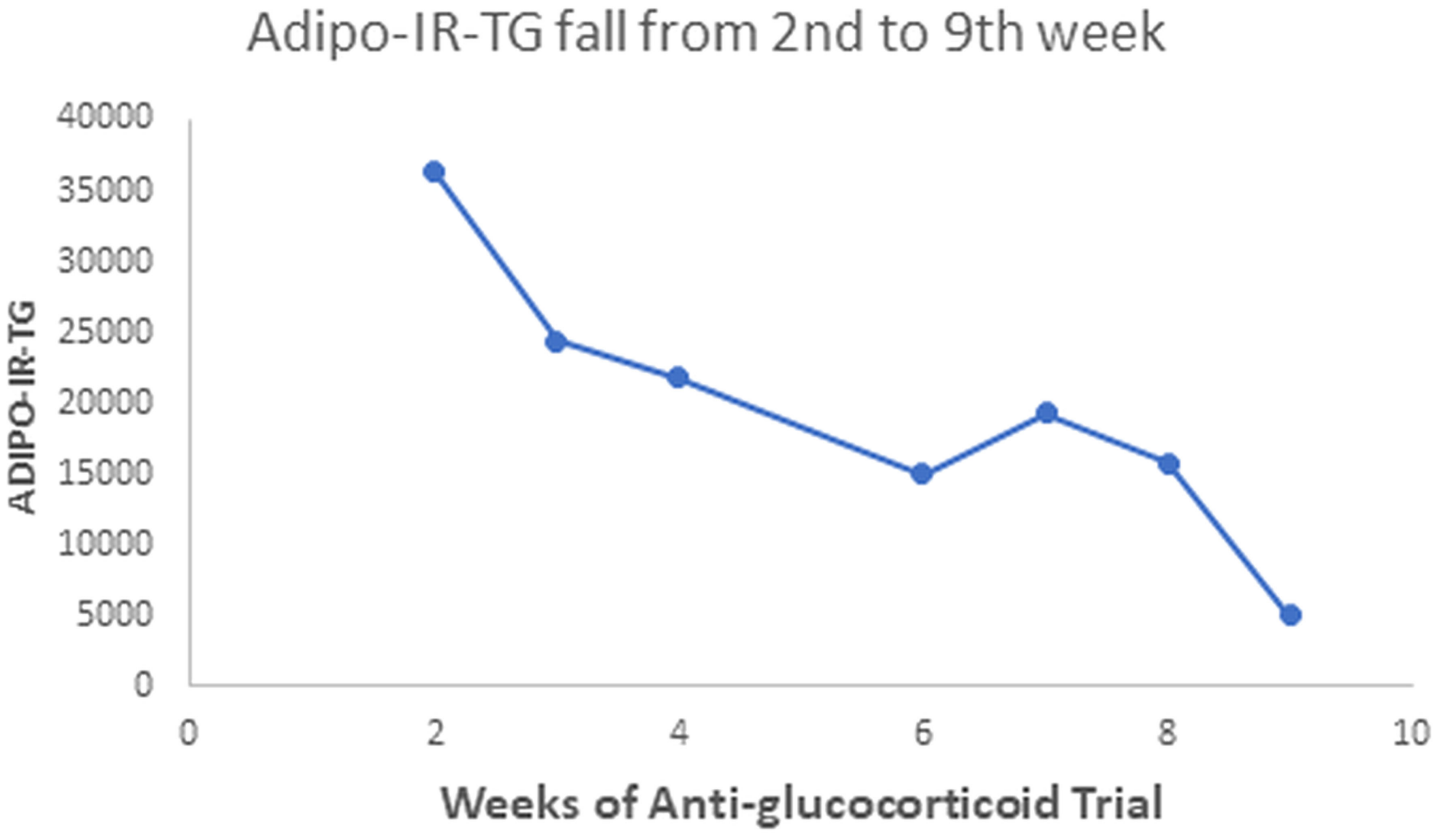
This Graph shows the fall in Adipo-IR-TG values from the second to the ninth week with more detail. These values fell from 36,250 to 4,936 mU/L*mg/dL.

**Figure 7:**
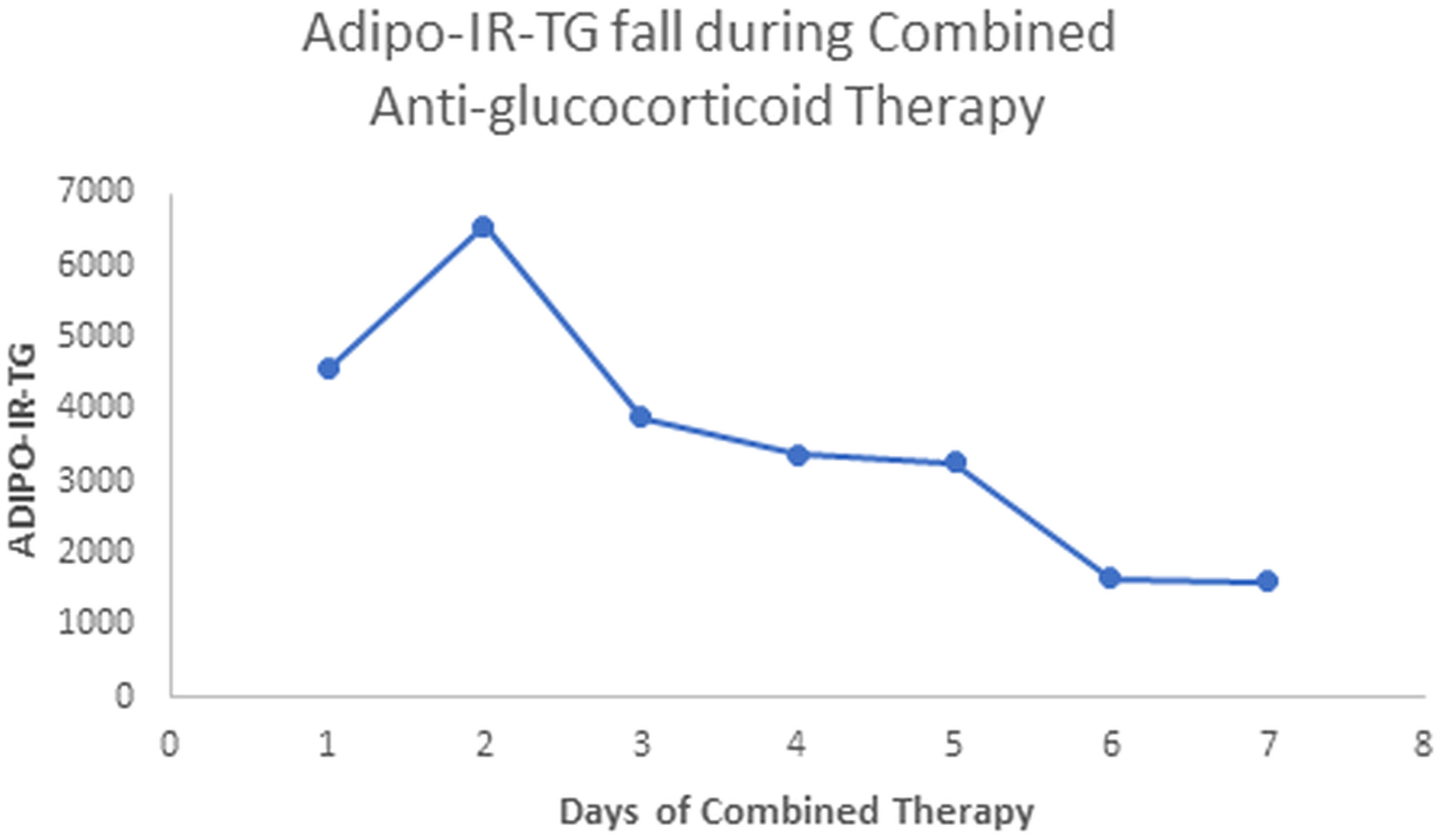
Here, we show the fall in Adipo-IR-TG values in week ten, from days 1 to 7 (Mifepristone plus Ketoconazole combined treatment). These values fell from 4,560 to 1,610 mU/L*mg/dL (probably acceptable values, up to 600 mU/L*mg/dL).

Our patient with Berardinelli-Seip syndrome at baseline had a sky-high Adipo-IR-TG level of 2,960,000 mU/L*mg/dL, as seen in **Figure 5**. After two weeks with Mifepristone alone, this level dropped to 36,250 (**Figure 6**). At the end of the nine weeks with Mifepristone alone, the Adipo-IR-TG dropped to 4,936. One week after adding Ketoconazole to reduce the elevated serum cortisol level, this level dropped further to 1,610 (**Figure 7**). So, it was clear that –by negating cortisol action– adipose insulin resistance (estimated with the Adipo-IR-TG) fell dramatically, from a maximal level of 2,960,000 to a minimum level of 1,610 mU/L*mg/dL, close to three times the upper limit in control subjects (600 mU/L*mg/dL).

In a recent study, the NIH group led by LK Niemann gave low-dose Mifepristone (50 mg every 6 hours for nine days) to sixteen overweight or obese subjects with prediabetes or mild Type 2 diabetes (22). These insulin-resistant subjects experienced an improvement in their adipocyte insulin sensitivity. Their adipose insulin resistance index (fasting serum insulin times serum FFA) had a 31.2% reduction at the end of the Mifepristone administration. Their hepatic insulin resistance index also fell after Mifepristone administration without reaching statistical significance. Finally, muscle insulin resistance –as estimated with the frequently sampled intravenous glucose tolerance test (FSIVGTT), the Matsuda index, and the Oral Glucose Insulin Sensitivity Index (OGIS)– did not change after Mifepristone administration.

In summary, a mild anti-glucocorticoid intervention demonstrated a beneficial reduction in adipose insulin resistance in insulin-resistant obese patients, paralleling the results observed in our CGL patient (using 600 mg of the anti-glucocorticoid drug). Therefore, Mifepristone alleviates adipose insulin resistance in both “adipocyte-replete” (obesity) and “adipocyte-depleted” (CGL) situations. Consequently, it is rational to infer that cortisol aggravates adipocyte lipolysis in these two conditions.

## 8 Assembling the puzzle of the pathogenesis of CGL

**Figure 8:**
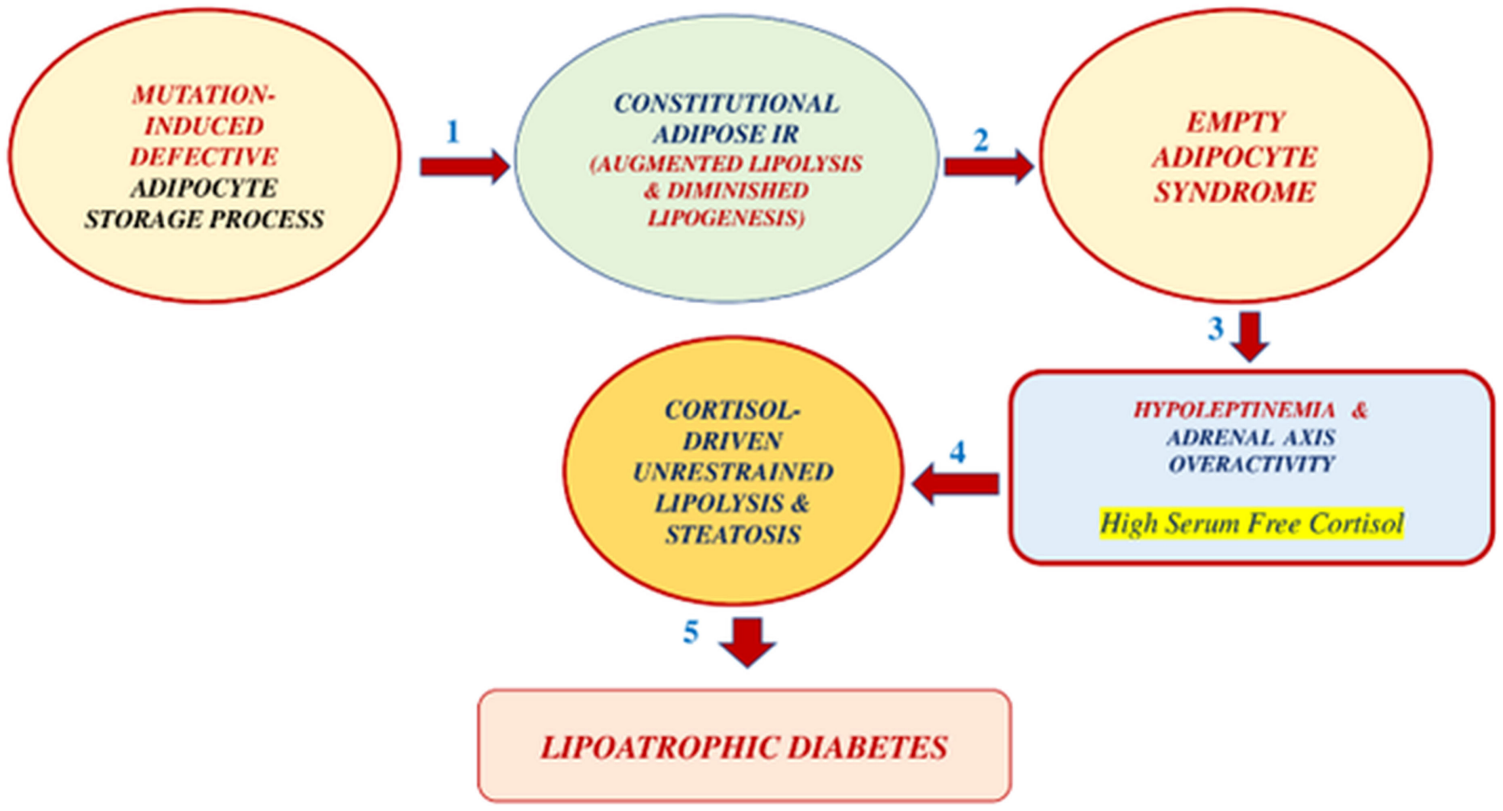
Proposed pathogenesis of CGL in 5 steps: 1. Constitutional adipose insulin resistance secondary to congenital defective adipocyte triglyceride storage. This defect leads to unrestrained lipolysis and reduced lipogenesis. 2. This situation leads to an empty-adipocyte syndrome with severe hypoleptinemia. 3. The reduced leptin action results in an adrenal axis overactivity, presumably with high plasma free-cortisol levels. 4. The latter effect produces cortisol-driven, unrestrained lipolysis and lean organ steatosis. 5. Three factors, liver steatosis, muscle steatosis, and islet b-cell apoptosis, lead to grotesque insulin-resistant lipoatrophic diabetes.

CGL subjects are born with an autosomal recessive mutation, deranging the ability of the adipose tissue to store triglycerides. The gene mutation of the CGL could primarily derange adipogenesis, lipogenesis, or lipolysis. We hypothesize (**Figure 8**) that no matter what part of this process might be at fault, the likely result would be a fat tissue abnormally prone to cortisol-driven lipolysis. Over time, the adipocyte becomes severely depleted of triglycerides, leading to an “empty adipocyte syndrome.” A triglyceride-depleted adipocyte is almost unable to produce leptin and adiponectin. Hypoleptinemia, in turn, leads to either excessive cortisol secretion (humans) or excessive corticosterone secretion (rats and mice).

On the other hand, hyperinsulinemia reduces the hepatic production of corticosteroid-binding-globulin, further increasing fatty tissue local exposure to cortisol. The latter situation should lead to a further acceleration of lipolysis. We hypothesized that the adipocytes of our patient are intrinsically insulin-resistant, leading to steatosis of the lean organs (including islet beta-cells), hepatic and muscle insulin resistance, and beta-cell apoptosis. Liver steatosis induces higher rates of gluconeogenesis, and muscle tissue steatosis reduces insulin-mediated glucose uptake. Steatosis of the islet beta-cell induces apoptosis, reducing its capacity to produce insulin, and the stage is ready for the appearance of severely insulin-resistant Type 2 diabetes mellitus. However, a lipolysis-prone fat depot may not be present in all types of CGL. Further research should clarify this point.

Leptin replacement in CGL subjects should attenuate the secretion of cortisol by the adrenal gland. Reduced cortisol secretion should reduce lipolysis and lean organ steatosis, thereby inducing a metabolic amelioration. Both Mifepristone and Ketoconazole therapy will reduce adipocyte exposure to corticosteroid action. This effect leads to lesser degrees of excessive lipolysis and metabolic amelioration. Total adrenalectomy, followed by a post-operative limited corticosteroid replacement, should limit adipocyte exposure to cortisol action. This effect should reduce lipolysis, leading to metabolic improvement. Chronic anti-glucocorticoid therapy in patients with CGL is impractical given its high cost and undesirable side effects, such as hypermineralocorticism (high serum cortisol acting as aldosterone in the renal tubule) and adrenal hyperplasia. Adrenalectomy is a feasible therapeutic alternative, but it has the inconvenience of being irreversible.

So, the question is: Is there a medical alternative to leptin replacement in treating CGL? We think there is!

## 9 The oral nonpeptide acth blockers

Theoretically, the ability to block the ACTH action at the adrenal level exhibited by the emerging nonpeptide oral ACTH blockers is a logical alternative to leptin replacement in CCL treatment. These substances might become an alternative to leptin replacement, given that they can reduce the adrenal secretion of cortisol. If our hypothesis on the pathogenesis of the CGL holds up, limiting the adrenal capacity to synthesize cortisol with oral ACTH blockers should result in metabolic amelioration in CGL subjects. Only one oral, nonpeptide ACTH blocker is in development for human use, the CRN04894, produced by Crinetics in San Diego, California (23, 24). The drug, aimed at treating ACTH-dependent diseases (ACTH-dependent Cushing syndromes and adrenal hyperplasia), has a half-life of 24 hours. It can be given orally, once daily at night. Forty-nine human volunteers received either 40, 60, or 80 mg daily. On day nine of treatment, urine free-cortisol (UFC) diminished by 31%, 72%, and 75%, respectively (24). ACTH and CRN04948 compete for one receptor site of the adrenal melanocortin-2 receptor (MC2-R). The MC2-R is transferred from the endoplasmic reticulum to the adrenal cell surface by the Melanocortin-2 Receptor Accessory Protein (MRAP).

## 10 Is hyperleptinemia with leptin resistance a condition leading to a hyperactive adrenal axis?

**Figure 9:**
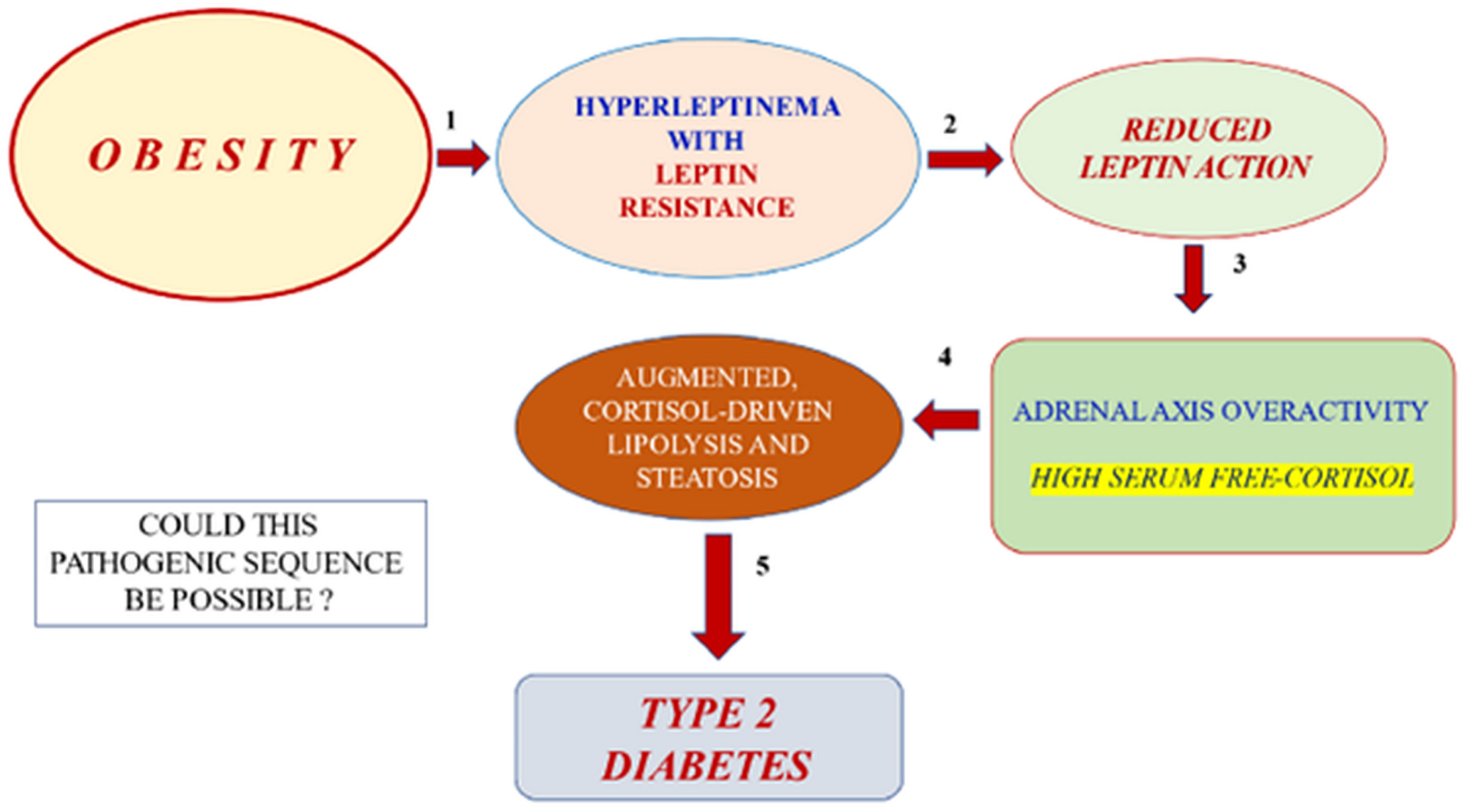
Hypothetical, possible pathogenic sequence (five steps) of obesity leading to Type 2 diabetes: 1. Obesity –with triglyceride-replete adipose cells– leads to hyperleptinemia and leptin resistance. 2. Leptin resistance leads to reduced leptin action. 3. The latter effect should result in adrenal cortex overactivity with elevated free cortisol levels in serum. 4. Enhanced corticosteroid exposure of adipose tissue should lead to excessive lipolysis (adipose insulin resistance) and peripheral lean organ steatosis (liver, muscle, and islet beta-cell).

The possibility of expanding the therapeutic alternatives for a nightmare disease such as CGL is gratifying and exciting. However, CGL is an ultra-rare disease. Therefore, we must use the new knowledge obtained in GCL studies to explore exciting therapeutic opportunities for common conditions such as obesity and Type 2 diabetes (**Figure 9**). Insulin resistance occurs when adipocytes store too much (obesity) or too little triglycerides (lipoatrophy). Excess triglyceride storage inside the adipocytes enhances leptin secretion. Hyperleptinemia, in turn, leads to leptin resistance, a condition of insufficient leptin action. A diminished leptin-mediated adrenal axis restraining should enhance cortisol-mediated lipolysis in obese subjects. CGL, on the other hand, is characterized by an empty adipocyte. This time, the expected cortisol-mediated lipolysis might be much worse than in obese subjects, given that adipose tissue in this condition might be inherently prone to lipolysis due to a mutation leading to a deficient triglyceride storage process.

In 1932, Harvey Cushing described the disease that bears his name (25). This disorder produces a grotesque clinical picture, with central obesity, arterial hypertension, dyslipidemia, and hyperglycemia. This picture reverts with the removal of the causing ACTH-producing pituitary adenoma. On the contrary, Addisonian subjects are lean and suffer from hypotension and hypoglycemia. So, cortisol excess is a prime suspect of provoking obesity, arterial hypertension, dyslipidemia, and hyperglycemia.

In 1988, Gerald Reaven described syndrome X, or insulin resistance syndrome (26). By far, the most common cause of insulin resistance is abdominal obesity. This latter clinical situation can be seen as a minor clinical manifestation of the endogenous Cushing’s disease, sharing insulin resistance, arterial hypertension, dyslipidemia, and glycemic abnormalities with the latest. Not surprisingly, many investigators thought central obesity with insulin resistance was secondary to some form of endogenous hypercortisolism. However, serum cortisol levels are either within normal limits or reduced in subjects with central obesity (27) while being high in Cushing’s syndrome. Nevertheless, the cortisol production rate is high in obese individuals (28). The explanation for this paradox (low serum cortisol levels with elevated cortisol production rates in obese individuals) is probably the low levels of Cortisol Binding Globulin (CBG) induced by hyperinsulinemia in these patients (29).

Excessive exposure of the adipocyte to serum cortisol in central obesity is due to increased cortisol production (with normal or reduced serum cortisol levels) and, additionally, to extracellular cortisone uptake and reactivation to cortisol.

The enzyme 11β hydroxysteroid dehydrogenase-1 (11βHSD-1) co-localizes with the glucocorticoid receptor inside the fat cell. This enzyme uptakes the inactive extracellular cortisone and activates it to cortisol. 11βHSD-1 is over-expressed in the subcutaneous and visceral adipose tissues of obese subjects (30). In short, obese subjects have high rates of cortisol production and metabolic clearance. They also have an elevated 11βHSD-1-mediated reactivation of extracellular cortisone to intracellular active cortisol. The lipolytic cascade of the fat cell is activated when exposed to higher serum free-cortisol levels. On the other hand, the enzymatic activity of 11βHSD-1 increases with glucocorticoids and leptin (31).

In obese people, the resistance to the antilipolytic effect of insulin seems secondary to the increased size of the fat cells rather than to inflammation of the fat tissue. Recently, investigators at the Mayo Clinic Endocrine Research Unit proved that a strong correlation exists between the concentration of serum insulin level producing a 50% inhibition of lipolysis (IC50) and human abdominal and femoral fat cell size. Indeed, after adjusting for adipose cell size, the univariate relationships between the adipose tissue inflammatory markers, senescent cells, IL-6, TNF-a mRNA, and IC50 lost significance (32). In short, obese subjects have high rates of cortisol production and metabolic clearance.

Let us agree that a reduced leptin action leads to an overactive adrenal axis. Therefore, obesity and diabetes-associated leptin resistance should be conditions with ACTH and cortisol excess. Both diseases are strongly associated with elevated adipose tissue insulin resistance and high fasting levels of circulating insulin and FFA.

However, the prevalence of elevated serum free-cortisol levels is unknown in these conditions.

Muraca et al., 2020, reported their findings on 884 obese subjects studied before subjecting them to bariatric surgery (33). A fraction of 129 patients (14.6%) had elevated UFC levels. One percent of the patients had elevated UFC and absent serum cortisol suppression after an overnight dexamethasone suppression test. Prediabetic (17.7%) and diabetic subjects (41%) exhibited a higher risk of having elevated UFC (OR 1.74 and 2.03, respectively). Among those with fasting euglycemia and normal HbA1c levels, those with high HOMA2 values had a higher risk of elevated UFC (OR 2.84). The authors concluded that in this obese population, both high HOMA2 values and dysglycemic conditions predicted hypercortisolism. In another provoking study, in 4,206 African American individuals from the Jackson Heart Study (34), 19% had prevalent Type 2 diabetes. A doubling of morning serum cortisol in non-diabetic subjects was associated with 2.7 mg/dL higher levels in fasting serum glucose and 10% lower HOMA β (insulin secretion). In comparison, among people with diabetes, a doubling of morning serum cortisol was associated with 23.6 mg/dl higher fasting serum glucose levels and 0.6% higher HbA1c. Among all participants, quartile four, compared with quartile one of serum cortisol, had 1.26-fold higher odds of prevalent Type 2 diabetes. In summary, much research is necessary to fully understand the issue of hypercortisolism associated with insulin- and leptin resistance.

Inside the adipocyte, cortisol stimulates the lipolytic enzymes, thereby increasing FFA and glycerol serum levels. Therefore, adipocytes exposed to increased local cortisol levels become insulin resistant. Excessive lipolysis should lead to steatosis in the liver, muscle, and islet beta-cells. Steatosis of these lean organs should translate into elevated liver gluconeogenesis, reduced muscle glucose uptake in response to insulin, and beta-cell apoptosis, leading to a declining pancreatic capacity to produce insulin.

Maybe a crucial concept in the pathophysiology of obesity –the leading cause of acquired insulin resistance– is that leptin resistance precedes insulin resistance. The reduced leptin action characteristic of the leptin resistance of obesity and Type 2 diabetes leads to a hyperactive adrenal axis. Therefore, restraining the adrenal axis should avoid the emergence of adipocyte insulin resistance. Without the latter phenomenon, liver, muscle, and islet beta-cell steatoses should not appear, thus blocking the road to Type 2 diabetes.

## 11 Concluding remarks

Berardinelli-Seip syndrome is an enlightening experiment of nature. Its pathogenesis is obscure, and its treatment is extraordinarily complex and expensive. Besides, leptin replacement is not available to most affected patients.

Even though we have no personal experience with Metreleptin in CGL, it has some drawbacks as a therapeutic option for this syndrome. This recombinant hormone has a high cost, difficult accessibility, and side effects, including hypoglycemia, weight loss, and the possible appearance of neutralizing antibodies to leptin (35). Our anti-glucocorticoid approach opens a window for new therapeutic alternatives to leptin replacement. Moreover, the effects of adrenalectomy in two mammals affected with CGL, one human patient and one transgenic mouse, allow us to understand better the complex pathogenesis of CGL. Two new therapeutic alternatives are alluring for affected patients and medical teams caring for them: total adrenalectomy and oral ACTH blockers. Adrenalectomy seems a helpful therapy for CGL, but it is an irreversible step that induces the fear of facing an adrenal crisis. Extended clinical experience has shown us that well-informed adrenalectomized patients rarely face an adrenal emergency since their bodies give them clear signs when they forget to take hormonal replacements. It seems more probable that a patient with Addison’s disease might face an adrenal crisis than an adrenalectomized patient. Of course, before exploring adrenalectomy in CGL patients, we should look for non-surgical alternatives. Therefore, we should explore the therapeutical potential of oral ACTH blockers on CGL subjects. Theoretically, their therapeutic use in CGL makes sense since they potentially mimic the leptin replacement effects on CGL. However, we must emphasize that the anti-glucocorticoid therapeutic approach might not necessarily benefit the different CGL types. Nevertheless, if anti-glucocorticoid treatment is proven successful in murine models of CGL, clinical investigators should explore its usefulness for every kind of CGL to test their therapeutic potential.

## Author contributions

PC and PV: bibliographic search, writing, data analysis, graphic design and review and/or revision of the manuscript. All authors contributed to the article and approved the submitted version.
